# Air pollutants and primary liver cancer mortality: a cohort study in crop-burning activities and forest fires area

**DOI:** 10.3389/fpubh.2024.1389760

**Published:** 2024-09-23

**Authors:** Natthapat Thongsak, Taned Chitapanarux, Anon Chotirosniramit, Somvilai Chakrabandhu, Patrinee Traisathit, Nawapon Nakharutai, Pimwarat Srikummoon, Salinee Thumronglaohapun, Titaporn Supasri, Phonpat Hemwan, Imjai Chitapanarux

**Affiliations:** ^1^Department of Statistics, Faculty of Science, Chiang Mai University, Chiang Mai, Thailand; ^2^Division of Gastroenterology, Department of Internal Medicine, Faculty of Medicine, Chiang Mai University, Chiang Mai, Thailand; ^3^Division of Hepatobiliary-Pancreatic Surgery, Department of Surgery, Faculty of Medicine, Chiang Mai University, Chiang Mai, Thailand; ^4^Division of Radiation Oncology, Faculty of Medicine, Chiang Mai University, Chiang Mai, Thailand; ^5^Atmospheric Research Unit of National Astronomical Research Institute of Thailand, Chiang Mai, Thailand; ^6^Geo-Informatics and Space Technology Centre (Northern Region), Department of Geography, Faculty of Social Sciences, Chiang Mai University, Chiang Mai, Thailand

**Keywords:** liver cancer, mortality rate, air pollutants, forest fire area, survival rate

## Abstract

**Introduction:**

Northern Thailand experiences high levels of air pollution in the dry season due to agricultural waste burning and forest fires. Some air pollutants can enter the bloodstream, and the liver has the role of detoxifying these along with other harmful substances. In this study, we assessed the effects of long-term exposure to air pollutants on liver cancer mortality in this area.

**Methods:**

A cohort of 10,859 primary liver cancer patients diagnosed between 2003 and 2018 and followed up to the end of 2020 were included in the study. Extended time-varying covariates of the annually averaged pollutant concentrations updated each year were utilized. The associations between air pollutants and mortality risk were examined by using a Cox proportional hazard model.

**Results:**

Metastatic cancer stage had the highest adjusted hazard ratio (aHR) of 3.57 (95% confidence interval (CI):3.23–3.95). Being male (aHR = 1.10; 95% CI: 1.04–1.15), over 60 years old (aHR = 1.16; 95% CI: 1.11–1.21), having a history of smoking (aHR = 1.16; 95%CI: 1.11–1.22), and being exposed to a time-updated local concentration of PM_2.5_ of 40 μg/m^3^ (aHR = 1.10; 95% CI: 1.05–1.15) increased the mortality risk.

**Conclusion:**

We found that air pollution is one of several detrimental factors on the mortality risk of liver cancer.

## 1 Introduction

Liver cancer is the sixth most common form of cancer after breast, lung, colorectal, prostate, and stomach cancer (1, 2). The World Health Organization (WHO) reported 905,677 new liver cancer cases in 2020, of whom 830,180 died (1, 2). Asia had the highest number of new cases (656,922; 72.5%), followed by Europe (87,630; 9.7%), and Africa (70,542; 7.8%) (1, 2). Of the Asian countries, Thailand had the second highest incidence of liver cancer (after Mongolia) with 27,394 cases, 26,704 of whom died, equating to an incidence rate of 39.2 and a mortality risk of 38.3 (1, 3). The World Health Organization estimates that the total number of new liver cancer cases in Thailand will increase to 42,600 by 2040, implying that the liver cancer incidence will persist and increase markedly within 20 years (4, 5).

People with hepatitis B and hepatitis C are known to develop cirrhosis, which can lead to liver cancer and accounts for 80% of all liver cancer cases worldwide (6, 7). Furthermore, it has been reported that diabetes increases the incidence and mortality of liver cancer (7–10). Moreover, nonalcoholic fatty liver disease (NAFLD), which can cause cirrhosis and thereby lead to liver cancer, is on the rise. In 2020, NAFLD patients had an incidence and mortality of liver cancer of 44 and 77 per 100,000 persons per year (11). Alcohol use is a long-established risk factor for liver cancer incidence and mortality (12–17), and even low-level consumption poses an approximately three-fold higher risk (18). Males exhibit a higher incidence of liver cancer compared to females, with studies consistently demonstrating a two to three times higher risk in in the former (14, 15, 17, 19). Usually, the lifestyle of men and women is different in both alcohol consumption and, especially, smoking (6, 12). Smoking is associated with a 30 to 70% increased risk of liver cancer compared to non-smokers (12, 20, 21).

Both *in vitro* and animal model studies have shown that exposure to PM_2.5_ causes oxidative stress in hepatocytes, leading to increased DNA damage and subsequent repair in the liver (22–25). This process can result in liver fibrosis similar to that seen in non-alcoholic fatty liver disease, which can exacerbate the initiation of liver cancer. Studies of humans have indicated that inhalation of pollutants can affect the liver through the circulatory system (26, 27). Brook et al. (28) reported an association between air pollutants and elevated serum levels of alanine transaminase (ALT), aspartate aminotransferase (AST), and gamma-glutamyl transpeptidase (GGT), which are known biomarkers for liver injury.

Particulate matter (PM) ≤ 2.5 μm (PM_2.5_) and ≤ 10 μm (PM_10_), nitrogen dioxide (NO_2_), sulfur dioxide (SO_2_), carbon monoxide (CO), and ozone (O_3_) are major air pollutants in Thailand (29). Thailand has consistently ranked among the top 50 countries with the highest ambient air pollution, particularly from 2019 to 2021 (30–32). Over the past decade, forest fires and agricultural waste burning during the dry season (January–April) have significantly reduced the air quality in northern Thailand (33). Thailand’s national annual average standards for PM_2.5_ and PM_10_ concentrations are 25 and 50 μg/m^3^, respectively, which are higher than those set by the WHO (10 and 20 μg/m^3^, respectively). According to the air quality measurement criteria used by IQ AIR (a Swiss organization), a PM_2.5_ level above 25 μg/m^3^ is slightly deleterious to health, above 35 μg/m^3^ is highly detrimental, and above 50 μg/m^3^ is hazardous (30).

In recent years, several research groups have investigated whether PM is associated with liver cancer mortality. The outcomes from a study in Taiwan indicate that exposure to PM_2.5_ levels greater than 36 μg/m^3^ is associated with a 58% increased risk of mortality from liver cancer, with each 5 μg/m^3^ increase in PM_2.5_ exposure being associated with a 13% increase (34). Meanwhile, another research group in Taiwan observed that a 1 μg/m^3^ increase in PM_2.5_ exposure is associated with an 11% increase in the mortality risk due to liver cancer (35). Similarly, the findings from another study conducted in California infer that exposure to PM_2.5_ after a diagnosis of hepatocellular carcinoma shortens life expectancy, with a 1 unit (5 μg/m^3^) increase in PM_2.5_ resulting in an 18% increase in mortality (36). From a US partial cohort study of non-smoking individuals (37), it was found that a 10 μg/m^3^ increase in PM_2.5_ is associated with a significant 2.18-fold increase in the risk of mortality from liver cancer.

Even though PM levels and liver cancer mortality rates appear to be related, the levels of PM_2.5_ and PM_10_ associated with the risk of mortality are still debatable and unconfirmed. In a recent systematic review (38), the authors reported that the outcomes from several studies suggest an association between PM_2.5_ level and liver cancer mortality but not with liver cancer incidence. Thus, we hypothesized that exposure to ambient air pollution after the development of liver cancer increases the risk of mortality. In prior studies, the researchers did not examine the effects of multiple air pollutants such as PM_2.5_, PM_10_, NO_2_, SO_2_, CO, and O_3_ over an extended period using time-varying covariates. Moreover, only a few researchers have explored the various causes of liver cancer within the socioeconomic context in Asia and compared this to other regions in the world. To the best of our knowledge, ours is the first cohort study conducted to examine the relationship between ambient air pollution, especially PM, and liver cancer mortality in northern Thailand, an area afflicted by crop-burning activity and forest fires for a significant part of the year. In addition, to the best of our knowledge, a cohort study to examine the relationship between ambient air pollution and liver cancer mortality in northern Thailand has not previously been conducted. Therefore, the aim of the present study is to investigate the effect of high levels of PM on the risk of mortality from liver cancer in this area.

## 2 Materials and methods

### 2.1 Study design and population

This retrospective cohort study was conducted to examine the mortality and associated risk factors on liver cancer patients in upper northern Thailand using extended time-varying covariates (PM_2.5_, PM_10_, NO_2_, SO_2_, CO, and O_3_ levels) over 15 years. Patients who were diagnosed with primary liver cancer [either hepatocellular carcinoma (HCC) or cholangiocarcinoma (CCA)] between January 1, 2003, and December 31, 2018, were followed up from their registered date to the end of 2020.

### 2.2 Exposure assessment for time-updated variables

The dataset used in the present study included concentrations of particulate matter, NO_2_, SO_2_, CO, and O_3_. Since these could have changed throughout the follow-up period, they were thus included as time-varying covariates in the analysis (39). Hourly air pollutant data from 2003 to 2020 were obtained from the Copernicus Atmosphere Monitoring Service (CAMS), the European Centre for Medium-Range Weather Forecasts (40, 41). The CAMS reanalysis merges modeled data utilizing a physics and chemistry-based atmospheric model with real observations to create a globally complete and consistent dataset consisting of 3-dimensional (3D) time-consistent atmospheric composition fields that include aerosols and chemical species (42). The spatial resolution for the dataset is approximately 80 km. The data are available in two formats of spectral coefficients: triangular truncation of linear grids (T255). The daily forecast beginning at 00 Universal Time Coordinated (UTC) for 48 h includes 3-hourly steps for the 3D model level and pressure level fields and hourly steps for the surface fields. In the present study, we utilized the average of the hourly concentrations to provide annual concentrations for each pollutant for each district in upper northern Thailand. These were then linked to the district of each patient’s residence (based on the assumption that their recorded address was where they lived and subsequently died) and the calendar year of diagnosis obtained from the Chiang Mai Cancer Registry. This was updated each year until the patient’s death, loss to follow-up, or censoring due to still being alive at the end of the study period.

### 2.3 Baseline and follow-up data

Information on each cancer patient at diagnosis, including demographics (gender, age, body mass index (BMI), smoking history, and alcohol-use history) and cancer characteristics [cancer stage (SEER staging; localized, regional, or metastatic)]. Each year, the concentration of each pollutant that each patient had been exposed to was obtained by using the pollution dataset detailed in previous section.

### 2.4 Statistical analysis

The baseline characteristics are presented as frequencies and percentages for categorical variables and medians and interquartile ranges (IQRs) for continuous variables. The follow-up time was calculated from the date of diagnosis to either the date of death regardless of the cause, to the last follow-up date, or censored by using the end of the study period (December 31, 2020), depending on which came first.

Missing values at the baseline of more than 30% for BMI, smoking history, and alcohol-use history were imputed using multivariate imputation based on linear regression for continuous variables and logistic regression for binary variables (43). In the context of this methodology, all missing values were substituted with imputed ones.

The overall rate of death and those separated by each variable were calculated as the number of deaths divided by the total number of persons per year of follow-up (PYFU). Confidence intervals (CIs) for the mortality risks were based on fitting the data to a Poisson distribution. Survival probability were obtained from Kaplan–Meier curves and log-rank tests were used to determine significant differences between the survival probabilities of the groups for each variable.

To handle these time-varying covariates, we used a time-dependent Cox proportional hazard model and a time-varying coefficient (44) to investigate the associations between the mortality risk among liver cancer patients and potential risk factors (gender, age, cancer stage, smoking history, alcohol-use history, calendar year of enrollment, and time-updated PM_2.5_, PM_10_, NO_2_, SO_2_, CO, and O_3_ concentrations). Log–log plots for survival and Schoenfeld residuals were used to verify the proportional hazards assumption for the Cox model for each covariate. All of the continuous variables were grouped by using quartiles and considered for dichotomization where appropriate [except for BMI with categories: < 18.5 and ≥ 18.5 kg/m^2^ (45)]. Factors associated with mortality risk with *p*-values <0.25 in the univariable analysis were included in the multivariable analysis via a backward elimination procedure, except for variables with high correlations (to avoid multicollinearity). All analyses were performed by using STATA (version 12).

## 3 Results

A total of 10,859 liver cancer patients were registered between January 2003 and December 2018, 7,763 (71%) of whom were male. At the time of diagnosis, the median age was 58.7 years old (IQR: 51.6–66.5) and the median BMI was 22.1 kg/m^2^ (IQR: 19.7–24.4). At the time of diagnosis, the medians for PM_2.5_, PM_10_, NO_2_, SO_2_, CO, and O_3_ levels were 37.5 μg/m^3^ (IQR: 33.5–42.1), 52.4 μg/m^3^ (IQR: 46.8–58.3), 7.2 ppb (IQR: 5.4–8.9), 5.8 ppb (IQR: 3.2–8.2), 390.4 ppb (IQR: 362.7–426.5), and 36.5 ppb (IQR: 35.0–38.5), respectively. Among the liver cancer patients, 6, 56, and 29% were diagnosed with the localized, regional, and metastatic cancer stages, respectively. In addition, 61% of the patients had an alcohol-use history while 54% had a smoking history. The median duration of follow-up and survival time were 1.0 years (IQR: 0.41–3.37) and 0.42 years (IQR: 0.17–1.24), respectively.

### 3.1 Baseline characteristics and mortality risk

According to the results in Table 1, 9,887 liver cancer patients died from any cause and the overall mortality risk was 68.0 per 100 PYFU. (95% CI: 66.7–69.4). The mortality risk was 72.4 per 100 PYFU for men (95% CI: 70.7–74.1) and 59.0 per 100 PYFU for women (95% CI: 56.9–61.2). Age at diagnosis ≥60 years had a high mortality risk (78.8 per 100 PYFU; 95% CI: 76.6–81.2), as did having a low BMI (79.3 per 100 PYFU; 95% CI: 75.5–83.4). When considering the three cancer stages, the mortality risk was highest in the group with the metastatic stage (179.6 per 100 PYFU; 95% CI: 173.4–186.1). Having a history of smoking and/or alcohol use also had high mortality risks (74.2 per 100 PYFU; 95% CI: 72.3–76.2 and 71.2 per 100 PYFU; 95% CI: 70.2–73.8, respectively).

**Table 1:** Baseline characteristics and the associated mortality risk of the study population.

Characteristic	Survived [*n* (%)]	Died [*n* (%)]	PYFU	Mortality risk^*^	95% CI
Overall	972 (9%)	9,887 (91%)	14,534	68.0	66.7–69.4
Gender					
Male	663 (8%)	7,100 (92%)	9,810	72.4	70.7–74.1
Female	309 (10%)	2,787 (90%)	4,724	59.0	56.9–61.2
Age at diagnosis (years old) [Median 58.7, IQR 51.6–66.5]					
< 60	571 (10%)	5,374 (90%)	8,808	61.0	59.4–62.7
≥60	401 (8%)	4,513 (92%)	5,726	78.8	76.6–81.2
BMI (kg/m^2^) [Median 22.1, IQR 19.7–24.4]					
< 18.5	116 (7%)	1,573 (93%)	1982	79.3	75.5–83.4
≥18.5	856 (9%)	8,314 (91%)	12,552	66.2	64.8–67.7
Cancer stage					
Localized	166 (27%)	453 (73%)	1731	26.2	23.9–28.7
Regional	631 (10%)	5,469 (90%)	9,443	57.9	56.4–59.5
Metastatic	46 (1%)	3,050 (99%)	1,698	179.6	173.4–186.1
Smoking history					
Yes	433 (7%)	5,414 (93%)	7,293	74.2	72.3–76.2
No	539 (11%)	4,473 (89%)	7,241	61.8	60.0–63.6
Alcohol-use history					
Yes	530 (8%)	6,106 (92%)	8,485	71.2	70.2–73.8
No	442 (10%)	3,781 (90%)	6,049	62.5	60.5–64.5

*Per 100 PYFU (persons per year of follow-up). CI, confidence interval; BMI, body mass index.

### 3.2 Survival probabilities

**Figure 1:**
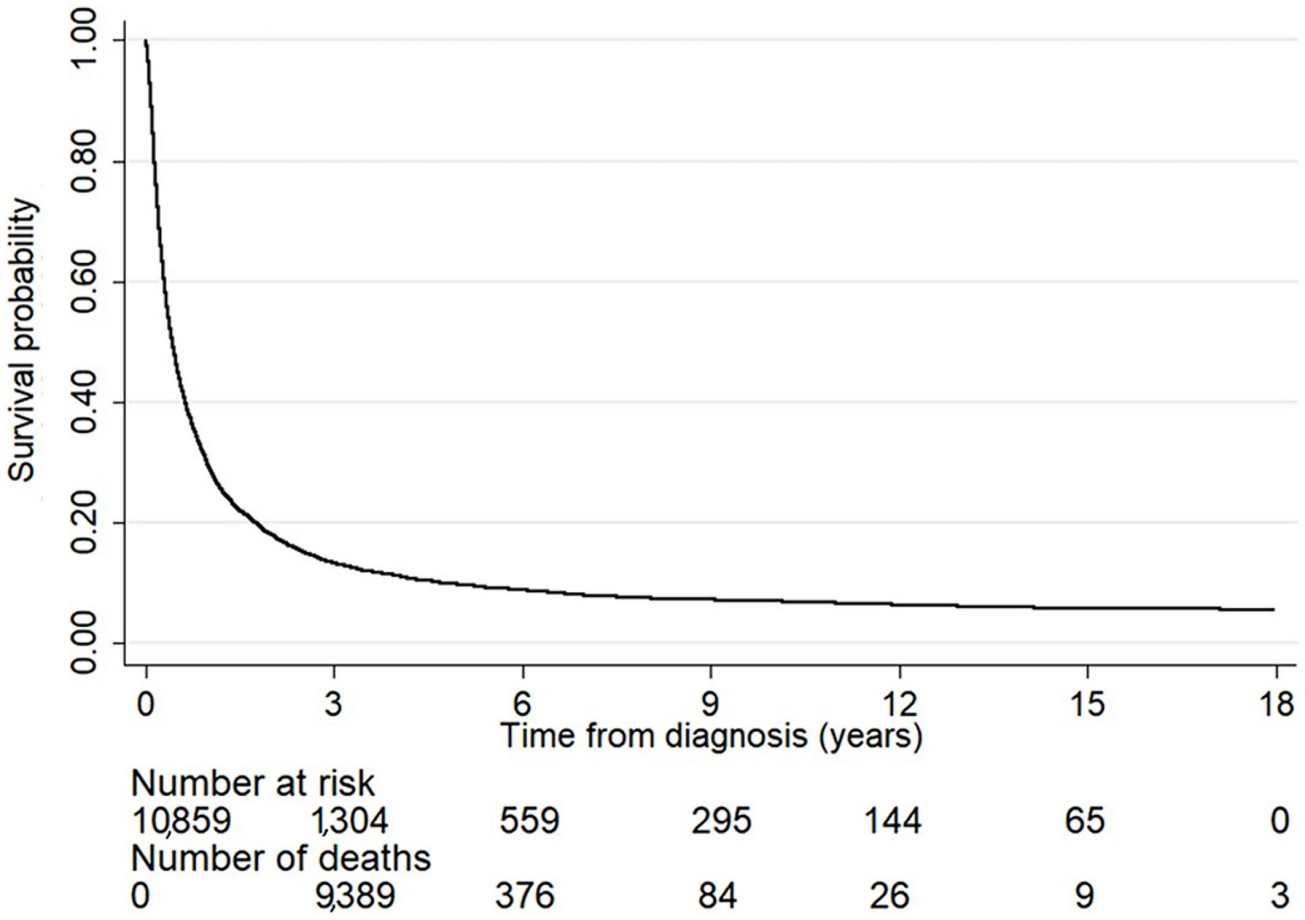
The survival probability of the liver cancer patients. The number at risk indicates the number of patients who were still alive each year after diagnosis. The number of deaths indicates the number of patients who died within the duration between a specific time point and the previous one. The overall survival probability sharply dropped within the first 3 years of diagnosis and slowly decreased after the follow-up time passed 3 years.

Figure 1 illustrates the overall survival probability of the liver cancer patients. Among them, 9,389 died within the first 3 years of diagnosis and the survival probability dropped sharply to 13%. This slowly decreased after a follow-up time of 3 years: the survival probabilities at 6, 9, and 12 years after diagnosis were 9, 7, and 6%, respectively, and thus the 5-year survival rate was estimated as 9.78%.

**Figure 2:**
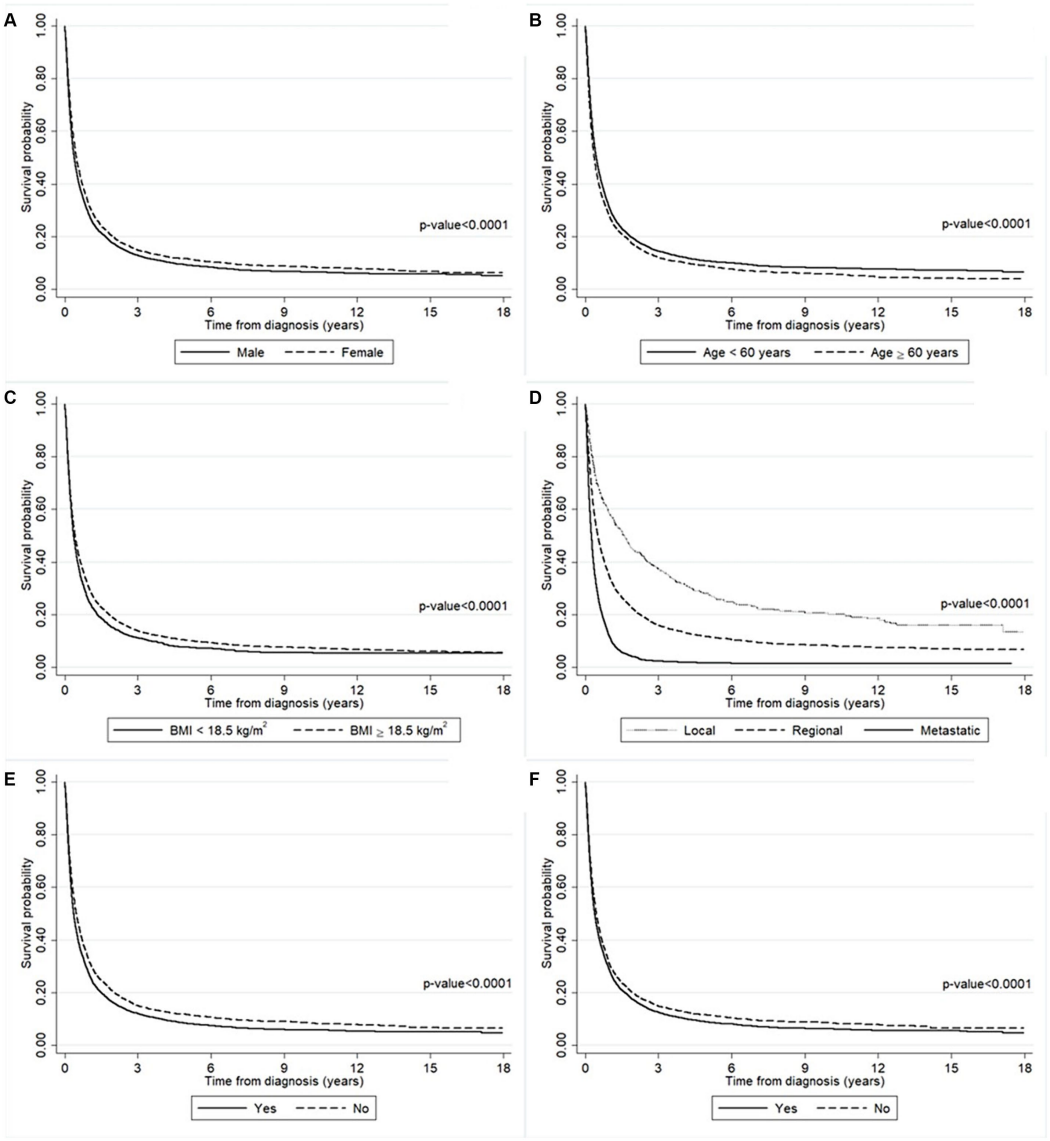
The survival probability of the liver cancer patients according to **(A)** gender, **(B)** age, **(C)** BMI, **(D)** cancer stage, **(E)** smoking history, and **(F)** alcohol-use history. The survival probability was significantly lower in the man group (solid line) than the women group (long-dashed line). The survival probability was significantly lower in the older group (long-dashed line) than the younger group (solid line). The survival probability was significantly lower in the lower BMI group (solid line) than the higher group (long-dashed line). The survival probability was significantly lower in the metastatic stage group (solid line) than the other groups (dashed lines). The survival probability was significantly lower in the smoking group (solid line) than the non-smoking group (long-dashed line). The survival probability was significantly lower in the drinking group (solid line) than the non-drinking group (long-dashed line).

The survival probability of the liver cancer patients according to the baseline characteristics including gender, age, BMI, cancer stage, smoking history, and alcohol-use history are presented in Figure 2. The results from log-rank tests show the differences between the survival probabilities of the groups for each variable (all *p*-values <0.0001). There is evidence that men had a significantly lower survival probability than women (Figure 2A). The results in Figure 2B suggest that liver cancer patients aged ≥60 years old had a significantly lower survival probability than younger ones. In addition, patients who had BMI < 18.5 kg/m^2^ had a significantly lower survival probability than those who had a higher BMI (Figure 2C). Liver cancer patients diagnosed with the metastatic stage had a significantly lower survival probability than those diagnosed with either the regional or localized stage (Figure 2D). Moreover, patients with a history of smoking and/or alcohol use had a significantly lower survival probability than non-smokers and non-drinkers (Figures 2E,F).

**Figure 3:**
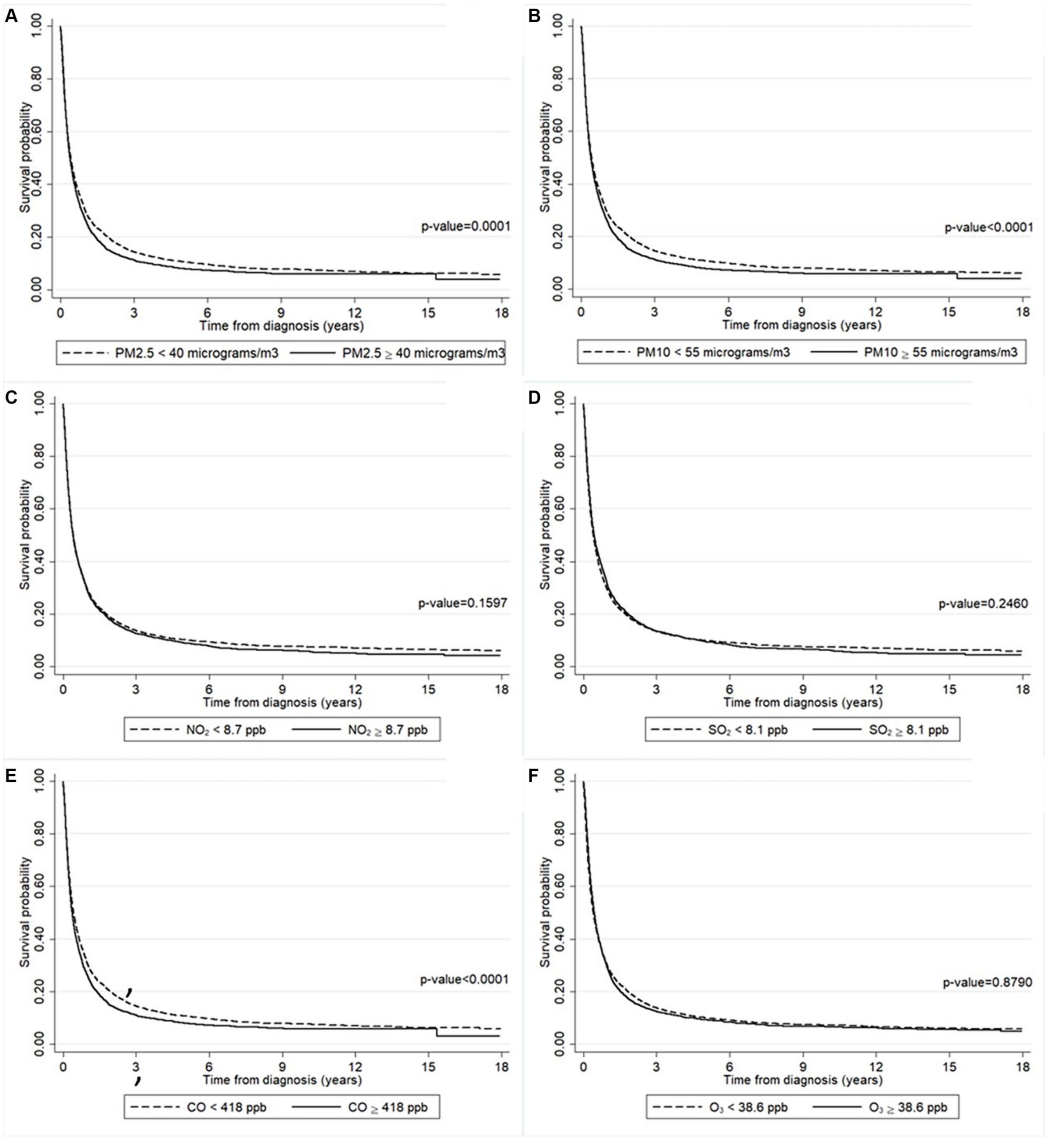
The survival probability of the liver cancer patients according to the annually averaged concentrations of **(A)** PM_2.5_, **(B)** PM_10_, **(C)** NO_2_, **(D)** SO_2_, **(E)** CO, and **(F)** O_3_. The survival probability was significantly lower in the patients who lived in a higher concentration of PM_2.5_ area (solid line) than those lived in a lower concentration of PM_2.5_ area (long-dashed line). The survival probability was significantly lower in the patients who lived in a higher concentration of PM_10_ area (solid line) than those lived in a lower concentration of PM_10_ area (long-dashed line). The survival probability was significantly lower in the patients who lived in a higher concentration of CO area (solid line) than those lived in a lower concentration of CO area (long-dashed line).

Figure 3 presents the effect of air pollution according to the patients’ residences on the survival probability. Figure 3A shows that patients who lived in an area where the annually averaged PM_2.5_ concentration ≥ 40 μg/m^3^ had a lower survival probability than where it was <40 μg/m^3^ (*p*-value = 0.0001). The same result was found for those who lived in an area where the annually averaged PM_10_ concentration ≥ 55 μg/m^3^ compared to where it was <55 μg/m^3^ (*p*-value <0.0001) (Figure 3B). Although not statistically significant, the survival probability of those who lived in an area where the annually averaged CO concentration ≥ 418 ppb was slightly lower than where it was <418 ppb (Figure 3E). Meanwhile, there were no differences in the survival probability of patients who lived in areas with varying concentrations of NO_2_ (Figure 3C), SO_2_ (Figure 3D), or O_3_ (Figure 3F).

### 3.3 Risk factors associated with death

Table 2 summarizes the results of Cox proportional hazard models for determining risk factors associated with the mortality risk among the liver cancer patients. The results from the univariable analysis show that gender, age, BMI, cancer stages, smoking history, alcohol-use history, and time-updated local concentrations of PM_2.5_, PM_10_, and CO were risk factors for death among the liver cancer patients (all *p*-values ≤0.0001).

**Table 2:** Risk factors associated with death among the liver cancer patients.

Characteristic			Univariable analysis			Multivariable analysis		
	Died	Total	HR	95% CI	*p*-value^*^	aHR	95% CI	*p*-value^*^
**At diagnosis**								
Male	7,100	7,763	1.11	1.07–1.17	<0.0001	1.10	1.04–1.15	< 0.0001
Aged ≥ 60 years old	4,513	4,914	1.13	1.08–1.17	<0.0001	1.16	1.11–1.21	< 0.0001
BMI < 18.5 kg/m^2^	1,573	1,689	1.12	1.06–1.18	<0.0001	1.06	1.00–1.12	0.0596
Regional cancer stage	5,469	6,100	1.78	1.61–1.95	<0.0001	1.80	1.64–1.99	< 0.0001
Metastatic cancer stage	3,050	3,096	3.46	3.13–3.82		3.57	3.23–3.95	
Smoking history	5,414	5,847	1.16	1.11–1.21	<0.0001	1.16	1.11–1.22	< 0.0001
Alcohol-use history	6,106	6,636	1.11	1.06–1.15	<0.0001	1.02	0.96–1.08	0.5924
**Time-updated variables**								
PM_2.5_ concentration ≥ 40 μg/m^3^	–	–	1.09	1.05–1.14	0.0001	1.10	1.05–1.15	< 0.0001
PM_10_ concentration ≥ 55 μg/m^3^	–	–	1.10	1.05–1.14	<0.0001	–	–	–
NO_2_ concentration ≥ 8.7 ppb	–	–	1.03	0.99–1.08	0.1614	–	–	–
SO_2_ concentration ≥ 8.1 ppb	–	–	0.97	0.93–1.02	0.2664	–	–	–
CO concentration ≥ 418 ppb	–	–	1.13	1.08–1.18	< 0.0001	–	–	–
O_3_ concentration ≥ 38.6 ppb	–	–	1.00	0.95–1.04	0.8792	–	–	–

*p-value from a partial likelihood ratio test. HR, hazard ratio; CI, confidence interval; aHR, adjusted hazard ratio.

Since there was a multicollinearity issue when including all of the pollutants in the multivariable model, we only retained PM_2.5_. Thus, the multivariable analysis included gender, age, BMI, cancer stage, smoking history, alcohol-use history, and the time-update local concentration of PM_2.5_. In the final model, we found that all of the included parameters were independently associated with the mortality risk (all *p*-values <0.0001), except for BMI and alcohol-use history. Especially, the metastatic cancer stage had the highest adjusted hazard ratio (aHR) = 3.57 (95% CI: 3.23–3.95). In addition, we also found that being male (aHR = 1.10; 95% CI: 1.04–1.15) and/or aged 60 years old (aHR = 1.16; 95% CI: 1.11–1.21), having the regional cancer stage (aHR = 1.80; 95% CI: 1.64–1.99) and/or a history of smoking (aHR = 1.16; 95% CI: 1.11–1.22), and/or being exposed to a time-updated local concentration of PM_2.5_ 40 μg/m^3^ (aHR = 1.10; 95% CI: 1.05–1.15) all increased the mortality risk among the liver cancer patients.

We checked for interactions between the variables included in the multivariable model (i.e., gender, age, BMI, cancer stage, smoking history, and alcohol-use history) and only found an interaction between BMI and cancer stage. However, we were unable to examine any interactions of these variables with the time-updated PM_2.5_ level.

## 4 Discussion

We are the first to study the survival probability and risk factors associated with liver cancer in upper northern Thailand using extended time-varying covariates (PM_2.5_, PM_10_, NO_2_, SO_2_, CO, O_3_ levels) based on a retrospective study over 15 years to answer this research. The survival probability of liver cancer patients concerning various factors such as gender, age, BMI, cancer stage, a history of smoking and/or alcohol use, and air pollution levels were examined in this retrospective study comprising a total of 10,859 liver cancer patients diagnosed over 15 years in upper northern Thailand. The results show that the overall mortality risk was 68 per 100 PYFU and the median survival time was 0.42 years. Gender, age, BMI, cancer stage, and smoking and alcohol-use histories were all found to significantly affect the survival probability. Being male and/or ≥ 60 years old, and/or having a low BMI, metastatic cancer, and/or a history of smoking or alcohol use all resulted in a lower survival probability, as did high PM_2.5_, PM_10_, and CO levels.

The mortality rate among liver cancer patients in our study was noticeably high (87% within 3 years after diagnosis). Although we did not include the impact of air pollution on liver cancer mortality in other regions of Thailand, we suspect that exposure to much higher levels of air pollutants in the northern region increases the risk of liver cancer mortality. In a previous study on the mortality from cholangiocarcinoma in Thailand from 2009 to 2013 (46), the one-year mortality rate in the northern region was considerably higher than in the central, northeastern, and southern regions where the ambient air pollution is much lower. Thus, expanding our study to include these other areas should be undertaken.

The potential mechanisms for the association between PM_2.5_ and liver cancer mortality remain unclear. PM_2.5_ contains various toxic elements, including heavy metals and other carcinogens, which could trigger the development and progression of cancer. At the molecular level, the genotoxic effects of PM_2.5_ include defects in DNA repair and replication, as well as DNA mutation (47). At the cellular level, PM_2.5_ induces cell damage and apoptosis (48), as well as oxidative stress and inflammation (49). PM_2.5_ causes oxidative stress by producing oxidants and free radicals and consuming antioxidants and enzymes. Diesel exhaust particles have been shown to cause oxidative stress in rats that resulted in DNA damage, the creation of bulky DNA adducts, the triggering of apoptosis, and the upregulation of hepatic DNA repair (23, 50). Long-term exposure to ambient air pollution has been linked to the upregulation of ALT activity, a biomarker for human liver damage (51–53). ALT and other liver function biomarkers and inflammation, such as C-reactive protein and interleukin-6, are used to detect the occurrence of liver cancer (54, 55). Therefore, exposure to ambient PM_2.5_ conceivably contributes to the development of and mortality from liver cancer.

PM_2.5_-associated mortality could be the result of oxidative stress induced by PM_2.5_ on epithelial cells creating reactive oxygen species that can damage DNA, proteins, and lipids (56, 57). Another explanation is that PM_2.5_-induced inflammation leads to the production of chemokines and cytokines that promote angiogenesis, thereby enabling the spread of metastatic cells to distant tissues (58). Hence, the carcinogenic effects of PM could stem from defects in DNA repair function and replication (47).

The effects of oxidative stress due to air pollution have been reported in other biological systems (59). Its effects on the digestive system include inflammation of the gut lining epithelial cells and alterations of the immune response and gut microbiota (56, 60). These could be connected to aerosolized pollutants becoming trapped in the mucus and swallowed. It is also well known that exposure to air pollution can increase inflammation in the human body, which can increase the number of tumor-associated macrophages and predispose an individual to cancer (61). In addition, air pollution adversely affects biological aging, the nervous system, smooth muscles, and the immune system (62, 63).

In rats, intragastric exposure to diesel exhaust particles induces oxidative stress associated with DNA damage, bulky DNA adduct formation, induction of apoptosis, and the upregulation of hepatic DNA repair (23, 50). In humans, long-term exposure to ambient air pollution causes the upregulation of biomarkers such as ALT for liver damage (51–53) and C-reactive protein (CRP) and interleukin-6 (IL-6) for inflammation (54, 55). Therefore, exposure to ambient PM2.5 conceivably contributes to the development of liver cancer.

Most of the risk factors in this study impacted the survival rate, which is unsurprising since liver cancer has a poor survival incidence (6). Our study showed that 91% of the patients died within 1 year. The correlation between ambient air pollution exposure and liver cancer might have been confounded by the general health of these individuals. Most cancer patients have poor immune resilience and thus have a higher risk of opportunistic infections (64), which we did not account for. It has been reported that opportunistic infection, a family history of cancer, and high alcohol consumption all significantly impact the liver cancer survival probability (65). Moreover, limited access to advanced cancer detection methods and treatment are major causes of the poor liver cancer survival rate in northern Thailand (66). Thus, although our results demonstrate an association between PM_2.5_ and poor liver cancer survival probability, we cannot assume that the causation is only due to high PM_2.5_ exposure.

The inclusion of local PM, NO_2_, and O_3_ concentrations as time-varying covariates in the analysis is one of the present study’s greatest strengths. Including them means that we could more accurately assess their impact on liver cancer patient survival. In addition, using such pollutant data is appropriate because the patients’ information was hospital-based recorded, which patients usually visit for their sickness. Therefore, we can assume that patients mostly stayed in their habitats, and we can imply that patients had accumulatively consumed pollutants, resulting in having precisely long-term exposure to pollutant concentrations. Another strength of this work is that information on liver cancer patients was collected from a considerable number of patients (*N* = 10,859) for 18 years.

It may be necessary to address some of these identified risk factors to reduce the mortality risk of liver cancer patients. For example, efforts should be made to encourage people to stop smoking and consuming alcohol, especially those with a high risk of contracting liver cancer. To improve the survival probability, it may be advantageous to focus on early cancer detection and treatment rather than wait until the cancer is in the metastatic stage. In addition, mitigating environmental factors such as air pollution is crucial for reducing the mortality risk of liver cancer patients. Overall, addressing individual and societal level risk factors is required to effectively reduce the mortality risk of liver cancer. This means that early diagnosis of liver cancer and tobacco control may be more critical for the prognosis of liver cancer in northern Thailand.

The strength of this study is that it is the first in which the mortality risk and risk factors associated with liver cancer were examined in upper northern Thailand using extended time-varying covariates (PM_2.5_, PM_10_, NO_2_, SO_2_, CO, O_3_ levels) retrospectively over 15 years. However, this study still has some limitations. First, we were unable to access other important risk factors for liver cancer: for instance, viral hepatitis status and the amount of alcohol consumption. Not including the latter data could have potentially biased the results as they would have helped to shed light on why alcohol-use history was not significant in the multivariable analysis. Second, the focus of this study was on patients living in the northern region of Thailand, so generalization of the findings is not possible. Since we did not compare the mortality of liver cancer patients and risk factors in other areas and settings, larger and more diverse study populations incorporating these would help to confirm the findings from our study. Third, several variables in this study contained missing values. We handled this issue using multivariate imputation based on regression, which could have introduced bias in the results. This might have been mitigated by using the Multiple Imputation by Chained Equations approach. Next, we considered keeping the BMI, age at diagnosis, and air pollutant levels as categorical variables even though dichotomization of these and the continuous variables could have led to confounding. In addition, we focused on the all-cause mortality of the patients and did not include competing events such as loss to follow-up or death from other causes in the analysis. Thus, the mortality rates reported in this study might be biased due to competing events and should be viewed with caution. Finally, the annually averaged pollutant concentrations as time-varying covariates used in this study might not accurately reflect individual-level exposure to air pollution over time. Moreover, although most of the study participants probably resided in the study area due to cultural and occupational reasons, some may have moved or spent time in other areas with different pollution levels. Although biological measures such as pollutant levels in blood samples would have more accurately determined the levels of air pollutant exposure of the participants, these data are not available in Thailand. Thus, the interpretation of our results should be treated with caution. A further prospective study using more precise data including biological measures might provide more precise findings. Next, including all of the air pollutants as variables caused multicollinearity issues in the multivariable model. Thus, we only retained PM_2.5_ since it has previously been reported to have a significant association with liver cancer mortality (35, 38). Using a different statistical model capable of addressing the multicollinearity issue could help to uncover the cumulative effects of multiple air pollutants on liver cancer mortality. In addition, we only examined the effect of air pollution on people already diagnosed with liver cancer. A future investigation of the effect of long-term exposure to air pollution, especially during early life, on liver cancer incidence may provide more insights.

## 5 Conclusion

Based on this retrospective cohort study, we found an association between mortality risk and exposure to a time-updated local concentration of PM_2.5_ > 40 μg/m^3^ in liver cancer patients who lived in Northern Thailand. Being male, aged >60 years old, and having a history of smoking were also significant deleterious factors. These findings provide health information that will encourage policymakers to combat air pollution in this area. However, the interpretation of our results should be treated with caution and further prospective research is needed to confirm our findings.

## Data availability statement

The data analyzed in this study is subject to the following licenses/restrictions: The datasets generated during and/or analysed during the current study are available from the corresponding author on reasonable request. Requests to access these datasets should be directed to Imjai Chitapanarux, imjai.chitapanarux@cmu.ac.th.

## Ethics statement

The studies involving humans were approved by the Research Ethics Committee of the Faculty of Medicine at Chiang Mai University. The studies were conducted in accordance with the local legislation and institutional requirements. The ethics committee/institutional review board waived the requirement of written informed consent for participation from the participants or the participants’ legal guardians/next of kin because patient consent was waived due to anonymous data recorded in the present study.

## Author contributions

NT: Conceptualization, Formal analysis, Writing – original draft, Writing – review & editing. TC: Data curation, Resources, Writing – review & editing. AC: Data curation, Resources, Writing – review & editing. SC: Data curation, Resources, Writing – review & editing. PT: Conceptualization, Formal analysis, Writing – original draft, Writing – review & editing. NN: Writing – original draft, Writing – review & editing. PS: Formal analysis, Writing – review & editing. ST: Formal analysis, Writing – review & editing. TS: Data curation, Resources, Writing – review & editing. PH: Data curation, Resources, Writing – review & editing. IC: Conceptualization, Data curation, Funding acquisition, Resources, Writing – review & editing.
